# Sex differences in psychophysical and neurophysiological responses to pain in older adults: a cross-sectional study

**DOI:** 10.1186/s13293-015-0041-y

**Published:** 2015-11-16

**Authors:** Todd B. Monroe, John C. Gore, Stephen P. Bruehl, Margaret M. Benningfield, Mary S. Dietrich, Li Min Chen, Paul Newhouse, Roger Fillingim, BettyAnn Chodkowski, Sebastian Atalla, Julian Arrieta, Stephen M. Damon, Jennifer Urbano Blackford, Ronald L. Cowan

**Affiliations:** Vanderbilt Psychiatric Neuroimaging Program, Vanderbilt University School of Nursing, Vanderbilt University Institute of Imaging Science, Nashville, TN USA; Vanderbilt University Institute of Imaging Science, School of Medicine, Nashville, TN USA; Vanderbilt University School of Medicine, Nashville, TN USA; Vanderbilt Psychiatric Neuroimaging Program, School of Medicine, Vanderbilt University Institute of Imaging Science, Vanderbilt University School of Medicine, Nashville, TN USA; Vanderbilt Center for Cognitive Medicine, Vanderbilt University School of Medicine, Nashville, TN USA; University of Florida Pain Research and Intervention Center of Excellence, University of Florida College of Dentistry, Gainesville, FL USA; Vanderbilt Memory and Alzheimer’s Center, Vanderbilt University Institute of Imaging Science, Nashville, TN USA

**Keywords:** Functional MRI, Pain, Older adults, Sex differences, Psychophysics, Thermal pain, Neuroimaging

## Abstract

**Background:**

Neuroimaging studies in younger adults have demonstrated sex differences in brain processing of painful experimental stimuli. Such differences may contribute to findings that women suffer disproportionately from pain. It is not known whether sex-related differences in pain processing extend to older adults.

**Methods:**

This cross-sectional study investigated sex differences in pain reports and brain response to pain in 12 cognitively healthy older female adults and 12 cognitively healthy age-matched older male adults (age range 65–81, median = 67). Participants underwent psychophysical assessments of thermal pain responses, functional MRI, and psychosocial assessment.

**Results:**

When compared to older males, older females reported experiencing mild and moderate pain at lower stimulus intensities (i.e., exhibited greater pain sensitivity; Cohen’s *d* = 0.92 and 0.99, respectively, *p* < 0.01) yet did not report greater pain-associated unpleasantness. Imaging results indicated that, despite the lower stimulus intensities required to elicit mild pain detection in females, they exhibited less deactivations than males in regions associated with the default mode network (DMN) and in regions associated with pain affect (bilateral dorsolateral prefrontal cortex, somatomotor area, rostral anterior cingulate cortex (rACC), and dorsal ACC). Conversely, at moderate pain detection levels, males exhibited greater activation than females in several ipsilateral regions typically associated with pain sensation (e.g., primary (SI) and secondary somatosensory cortices (SII) and posterior insula). Sex differences were found in the association of brain activation in the left rACC with pain unpleasantness. In the combined sample of males and females, brain activation in the right secondary somatosensory cortex was associated with pain unpleasantness.

**Conclusions:**

Cognitively healthy older adults in the sixth and seventh decades of life exhibit similar sex differences in pain sensitivity compared to those reported in younger individuals. However, older females did not find pain to be more unpleasant. Notably, increased sensitivity to mild pain in older females was reflected via less brain deactivation in regions associated with both the DMN and in pain affect. Current findings elevate the rACC as a key region associated with sex differences in reports of pain unpleasantness and brain deactivation in older adults. Also, pain affect may be encoded in SII in both older males and females.

**Electronic supplementary material:**

The online version of this article (doi:10.1186/s13293-015-0041-y) contains supplementary material, which is available to authorized users.

## Background

Pain is the primary reason that people seek medical attention [1]. Chronic pain affects one-quarter of the world’s population, and the prevalence of chronic pain increases with age [2]. This increased risk of pain in aging appears to be compounded by age-related declines in the function of endogenous pain inhibitory systems [3–5]. In addition to the increased risk of pain associated with aging, sex differences in pain risk have been reported [6], with women generally reporting a higher prevalence of chronic pain that increases with age [7, 8]. In a large-scale study of pain reports in males and females, females reported higher pain intensity scores than males for comparable pain conditions [9].

**Fig. 1:**
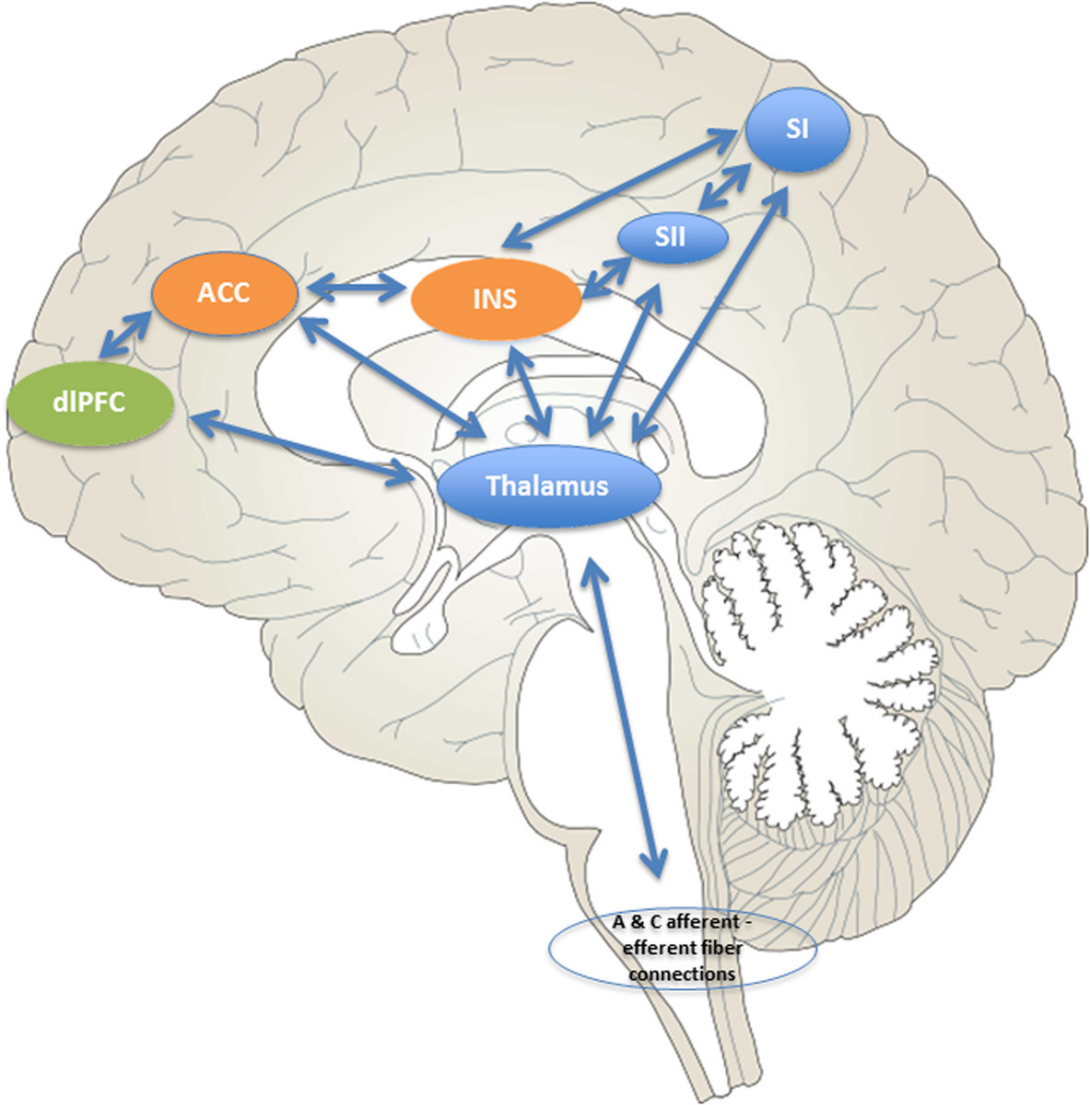
Basic pain matrix model. Basic structures of the “pain matrix.” *Blue areas* represent common structures in the sensory/discriminative (lateral) pain pathway (thalamus, SI, SII). *Orange areas* represent common structures identified in the affective/motivational (medial) pain pathway (ACC, INS). The *green region* represents one component of the cognitive/evaluative pain system (dlPFC). The *arrows* represent multiple cortical connections between regions and systems indicating the complex interconnectedness of brain regions involved with pain. *ACC* anterior cingulate cortex, *INS* insula, *dlPFC* dorsolateral prefrontal cortex, *SI* primary somatosensory cortex, *SII* secondary somatosensory cortex

Central pain processing is classically described in basic neural circuits comprising the “pain matrix” consisting of the sensory/discriminative, affective/motivational, and cognitive/evaluative networks [10–13]. In brief, the sensory/discriminative (sometimes referred to as the lateral) pathway includes the ventral posterolateral (VPL), thalamic nucleus, and primary (SI) and secondary (SII) somatosensory cortices. The affective/motivational and cognitive/evaluative components of pain are typically included in the (medial) pathway and include the dorsomedial (DM) thalamic nucleus, the dorsolateral prefrontal cortex (dlPFC), the insula (INS), and the anterior cingulate cortex (ACC; Fig. 1). While the INS cortex is generally included in the affective/motivational pathway, the role of the INS in salience detection and executive function is quite complex [14] and should deservedly be described with functions in multiple pain networks. The anterior INS (aINS) is associated with relaying affective information [15], while the posterior INS (pINS) is associated with the detection of pain sensation and discrimination [16]. Emerging evidence from human neuroimaging studies suggests that a much broader array of brain regions than those classically included in the “pain matrix” are important to the pain experience and that these pain networks do not function independently [17–19].

In addition to the abovementioned sex differences in the clinical experience of pain, psychophysical studies of responses to experimental thermal pain stimuli in younger adults suggest sex differences. In these studies, females generally exhibit lower pain threshold and lower pain tolerance levels, while commonly rating pain to be more intense and more unpleasant than do males (reviewed in [6]). Functional neuroimaging studies examining the neurophysiological basis of sex differences in pain processing have generally been restricted to younger cohorts (i.e., those under 65 years of age). These studies have revealed a variety of sex-related differences in brain activation during experimental pain delivery [20, 21]. A recent study found that in younger adults, functional connectivity (FC) differed by sex with women demonstrating greater FC between the subgenual ACC (sgACC) and several regions modulating descending pain, whereas men demonstrated greater FC between the sgACC and several regions implicated in sustained attention to pain, potentially delaying descending modulation [22].

Since there are limited reports of sex-associated differences in the psychophysical and neurophysiological processing of pain in older adults (i.e., those 65 years of age and older), the aim of the current study was to examine sex differences in both psychophysical (as measured with verbal pain reports) and neurophysiological (as measured by brain activation) responses to standardized (perceptually matched) experimental thermal heat pain in healthy older adults. Based on existing literature in younger cohorts, our first hypothesis (psychophysical) was that, when compared to older males, older females would report “mild” and “moderate” pain at lower stimulus intensities (i.e., be more pain sensitive) and would report greater perceived pain unpleasantness even with no sex difference in perceived pain intensity. Our second hypothesis (neurophysiological) was that, when compared to older males, older females would exhibit greater brain activations in the sensory/discriminative, affective/motivational, and cognitive/evaluative pain networks in response to experimental thermal pain delivery.

## Methods

This cross-sectional study was approved by the Vanderbilt University Institutional Review Board, and each participant provided written informed consent at the time of enrollment. From an ongoing study on pain in dementia, 24 age-matched healthy volunteers (12 men, 12 women) aged 65 to 81 years with a median age of 67 years were selected for the analyses of sex differences.

### Screening and enrollment of participants

Participants were recruited from the Nashville, Tennessee, metropolitan area using mass email, flyers, and recruitment presentations in facilities (e.g., assisted living facilities, retirement homes, and adult day care services) and at local events such as healthy aging seminars. A two-part screening process included an initial telephone screening followed by a 1-h consenting/enrollment visit scheduled at the participant’s place of residence (e.g., home or independent living facility). During the consenting/enrollment visit, a trained research assistant verified inclusion and exclusion criteria. Participants were excluded for the following reasons: presence of chronic pain diagnosis, cognitive impairment (score ≤28 on Mini-Mental State Exam (MMSE) [23]), regular use of opioid or non-opioid pain medication, history of stroke, cancer, peripheral neuropathy, Raynaud’s Disease, unstable cardiac conditions, insulin dependent diabetes, hormone replacement therapy (females), or a current diagnosis of major depression. Magnetic resonance imaging (MRI) exclusion criteria were claustrophobia; presence of pacemaker, ventricular shunt, or any implanted metal object that could not be confirmed as 3 Tesla (3 T) MRI compatible; multiple metal implants in the same extremity; or presence of movement disorders such as Parkinson’s disease, restless leg syndrome, or essential tremor. Since hormone replacement therapy has been shown to increase experimental pain thresholds in females [24], we excluded participants who were prescribed hormone replacement therapy. Socioeconomic status (SES) may also impact the experience and reporting of pain [25]; therefore, the groups were matched on SES. All participants were instructed to avoid drinking caffeine for 4 h before scanning and not to use any pain medication (opioid or non-opioid) for at least 24 h prior to data collection. Participants were reimbursed $100.00 for their time.

### Assessments

Participants underwent 1 h of psychosocial assessments during the home visit. These included a detailed list of all regularly scheduled and *pro re nata* medications, demographic information, Hollingshead Four-Factor SES [26], MRI safety clearance, and cognitive screening with both the MMSE [23] and the dementia rating scale (DRS) [27]. On the day of the MRI procedures, each subject was further assessed with the Brief Pain Inventory (BPI) [28], Geriatric Depression Scale (GDS) [29], and State-Trait Anxiety Inventory (STAI) [30].

### Thermal stimulation protocol (psychophysics)

Since the current study was part of a larger study on pain in people with and without dementia, the thermal stimulation protocol used in this study was a modification of the experimental mechanical pressure pain protocol used successfully by Cole and colleagues to examine psychophysical responses and brain activations in people with Alzheimer’s disease [31]. Upon arrival at the Vanderbilt University Institute of Imaging Science, participants underwent a thermal pain psychophysics evaluation (~30 min) followed by one MRI session (~50 min). Pain psychophysics were assessed using the Medoc Pathway Pain and Sensory Evaluation System [32] in a room adjacent to the MRI scanner. The Medoc thermode (30 × 30 mm) was attached to the thenar eminence of the right hand.

Before beginning sensory threshold testing, participants were told, “There are two aspects of pain which we are interested in measuring: the intensity, how strong the pain feels, and the unpleasantness, how unpleasant or disturbing the pain is for you” [33]. Next, participants were shown a 0–20 sensory pain intensity scale used in prior work [32], which included the anchors “warmth = 0,” “mild pain = 5,” and “moderate pain = 11.” Additionally, each participant was read the following: “I will tell you when the metal cube that is attached to your hand will start heating up, then I will ask you to stop the heat when you feel ‘warmth,’ ‘mild pain,’ or ‘moderate pain.’ I will not ask you to rate any pain greater than ‘moderate pain.’ An example of ‘mild pain’ might be the temperature of a hot bath and an example of ‘moderate pain’ might be the temperature of a fresh hot cup of coffee.” Next, participants were shown a parallel 0–20 unpleasantness scale with the following anchor descriptions: “0 = neutral,” “5 = slightly unpleasant,” “8 = unpleasant,” “11 = very unpleasant,” “16 = intolerable,” and “20 = extremely distressing” [32]. Instructions given for these ratings were “After you stop the heat, I will ask you to tell me how unpleasant the previous temperature was.”

We defined the baseline temperature as 30 °C (a temperature not perceived as warm or cold [34]) and programmed the thermode to deliver heat increasing at a rate of 4 °C/s. We modeled our thermal stimulus delivery after Wager and colleague’s successful paradigm in which each instance of a temperature began from baseline and ramped up and down at a moderate rate [19]. Subsequently, we recorded the temperature at which each participant reported the sensations of warmth, mild pain, and moderate pain. Each participant completed three trials at each temperature condition. The average temperature (in degrees Celsius) across the trials at each level of intensity was used in analyses (max temperature = 48 °C). Immediately after indicating the first stimulus meeting criteria for each of the three intensity levels, participants were asked to rate the unpleasantness associated with that stimulus level (i.e., warmth, mild pain, and moderate pain) as described above.

### Brain imaging acquisition: structural and functional

Brain images were acquired on a Philips 3T Achieva MRI scanner (Philips Healthcare Inc., Best, Netherlands). A standard whole-brain 3-D anatomical T1-weighted/time of flight echo (TFE with SENSE coil) scan was acquired. In each 264-s-duration functional run, 28 field echo planar imaging (EPI) (162 dynamics, 4.50-mm slice thickness with 0.45-mm gap, 2-s time to repeat (TR), 35-ms echo time (TE), 79° flip angle, field of view (FOV) = 240, matrix = 128 × 128) scans were acquired.

### Thermal stimulation protocol (fMRI)

**Fig. 2:**
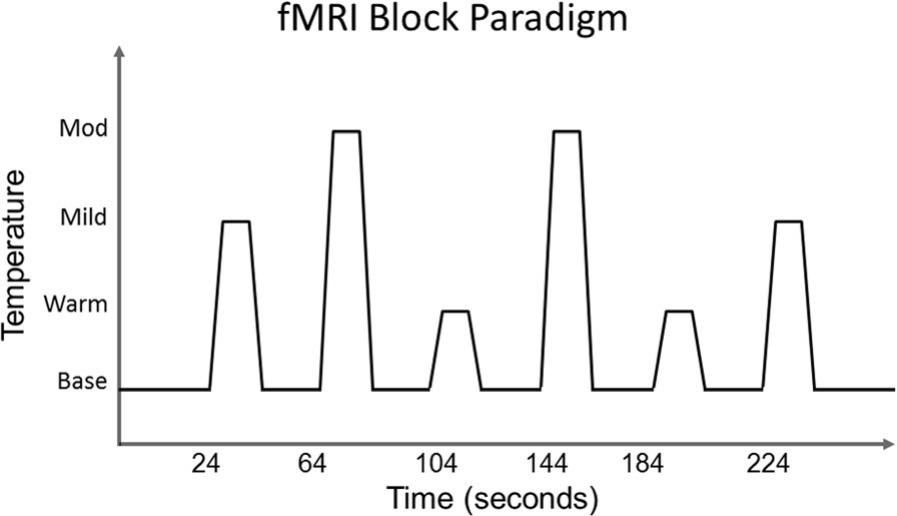
fMRI paradigm. Graphical depiction of experimental paradigm design demonstrating timing of pseudo-randomly delivered thermal stimuli during four functional MRI scans. Not to scale. Scan time 264 s per functional run

During functional MRI (fMRI), heat pain stimulation was administered to the thenar eminence of the right hand using the same Medoc pathways system described above. Prior to scanning procedures, the Medoc was programmed with each participant’s average temperature rated as producing warmth, mild pain, and moderate pain percepts derived during the pain psychophysics testing session. An fMRI block design with six thermal stimulation periods (two at each intensity; duration 16 s each) followed by baseline (30 °C) periods (duration 24 s) was used (Fig. 2). To avoid order effects, each thermal condition was pseudo-randomly delivered two times over four functional runs. During each functional run, lights remained on and participants were instructed to be as still as possible and to remain awake with eyes open. After each functional run, study personnel verbally communicated with each participant to confirm alertness and comfort with study continuation. Visual and audio contact was maintained during all scanning procedures.

### Image processing

Slice timing correction and motion correction (using standard rigid body registration of intra-scan volumes) were applied to the fMRI data using standard SPM8 techniques. Using the first image volume from each fMRI imaging run, volumes were co-registered to structural T1-weighted volumes. Images were spatially smoothed with an 8-mm full-width half-maximum (FWHM) Gaussian kernel. Structural data were registered to Montreal Neuroimaging Institute (MNI) space and the resulting transformation matrix was applied to the fMRI data.

### Data analysis

Whole-brain fMRI activation was modeled as the contrast of temperature stimuli (those temperatures perceived as producing warmth versus mild pain versus moderate pain against a fixed baseline). We also modeled the period required for the thermal probe to ramp up to target temperature and to ramp down from target temperatures. Because individual temperature threshold precepts were measured, ramp up and ramp down periods and individual temperature percepts were modeled as covariates of no interest in the general linear model (GLM). These resulting subject-specific contrast maps were used in higher-level analyses for between-group comparisons and within-group analysis in SPM8 to compare noxious pain (mild and moderate) versus innocuous warmth. These analyses generated an activation map of t-statistics that were used to identify brain regions indicating statistically significant between-group (male versus female) activation differences. To account for multiple comparisons, statistical thresholds for these higher-level analyses were corrected using the intrinsic smoothness of the data [35] and Monte Carlo simulations in 3dClustSim (http://afni.nimh.nih.gov/pub/dist/doc/program_help/3dClustSim.html) at 10,000 iterations to produce family wise error corrected data (*p* ≤ 0.05) based on whole-brain analysis with a cluster size of 1659 voxels for significance. After generating whole-brain statistically significant clusters, peak MNI coordinates were identified in pain regions of interest (ROIs) within those clusters. Using Marsbar [36], we created 3-mm spheres around select peak coordinates in pain processing regions and extracted the average signal for each ROI activated in males and females for each contrast/condition to use in analyses of the association of psychophysical reports with brain activation.

Demographic and standardized sample characteristics were non-normally distributed and summarized using median and 25th to 75th inter-quartile ranges (IQR; continuous data) and Ns (percentages; categorical data). Medians and IQR were also used to summarize the psychophysical and ROI percent signal change data. Mann-Whitney tests were used to test for sex differences in the self-reported temperature and unpleasantness ratings at each of the pain sensory levels, as well as difference in the amount of change in those self-reports between pain levels. Associations of psychophysics (temperature intensity and unpleasantness) changes with respective contrast signal change values in ROIs associated with pain were assessed using linear regression analyses. To match the signal change contrast conditions (e.g. mild versus warm), each analysis controlled for the temperature and unpleasantness ratings in the referent pain condition also (e.g. warm temperature or unpleasantness if the focus in on the mild versus warm signal change condition). Tests of differences between the groups in the strength of those regression coefficients were conducted using the *z* test for independent correlations. Because this study focuses on a subset of participants in an ongoing larger study and because it is a preliminary investigation of the phenomena, statistical powering for this specific study was not conducted, rather, the primary focus is on the effect sizes observed. Thus, effect indices (e.g., Cohen’s *d* and *beta* coefficients) are reported with respective statistical *p* values and if of sufficient magnitude to demonstrate promising potential for future research, may be interpreted even if the respective *p* value does not meet the statistical significance criteria. Those findings, of course, are interpreted with caution. For these same reasons, we did not use any type of correction to the alpha level used. Unless otherwise noted, *p* < 0.05 was used for determining statistical significance.

## Results

### Demographics

There were no statistically significant differences between females and males on average or current bodily pain (BPI [28]), depression (GDS [29]), state or trait anxiety (STAI [30]), cognitive status (MMSE [23]), or SES (Hollingshead Four-Factor SES [26]) scores (see Table 1; all *p* > 0.05).

**Table 1:** Demographic and clinical summaries by sex

	Total (*N* = 24)	Female (*N* = 12)	Male (*N* = 12)	*p* value
	Median (IQR)	Median (IQR)	Median (IQR)	
Age	66.5 (65,69)	67.0 (65,70)	66.5 (66,69)	0.724
Race	*N* (%)	*N* (%)	*N* (%)	0.218
Caucasian	21 (87.5)	10 (83.3)	11 (91.7)	
African-American	2 (8.3)	2 (16.7)	0 (0.0)	
Asian	1 (4.2)	0 (0.0)	1 (8.3)	
Marital status				0.206
Married	15 (62.5)	6 (50.0)	9 (75.0)	
Not married	9 (37.5)	6 (50.0)	3 (25.0)	
Marital occupational status				0.102
One spouse gainfully employed	12 (50.0)	8 (66.7)	4 (33.3)	
Both spouses gainfully employed	12 (50.0)	4 (33.3)	8 (66.7)	
Level of school completed				0.241
High school	3 (12.5)	3 (25.0)	0 (0.0)	
Technical/some college	8 (33.3)	3 (25.0)	5 (41.7)	
College graduate	5 (20.8)	3 (25.0)	2 (16.7)	
Advanced degree	8 (33.3)	3 (25.0)	5 (41.7)	
Standardized measures				
BMI^a^	27.4 (23,30)	25.1 (23,29)	28.1 (25,30)	0.133
Total SES score^b^	56.3 (45,66)	57.5 (35,64)	55.5 (45,66)	0.560
MMSE score^c^	30.0 (29,30)	30.0 (28,30)	30.0 (29,30)	0.641
BPI-SF average pain^d^	1.0 (0,2)	2.0 (0,3)	1.0 (0,2)	0.512
BPI-SF pain right now^d^	0.0 (0,1)	0.0 (0,0)	0.0 (0,1)	0.403
GDS-SF score^e^	0.0 (0,2)	0.0 (0,2)	0.5 (0,2)	0.425
STAI state score^f^	48.5 (45,51)	50.0 (45,53)	47.0 (44,50)	0.117
STAI trait score^f^	47.5 (45,51)	49.5 (46,53]	46.5 (43,49]	0.068

^a^BMI = body mass index

^b^Hollingshead Four-Factor Measure of Socioeconomic Status (range = 8–66; 8 = lowest SES, 66 = highest SES)

^c^MMSE-Folstein Mini-Mental State Examination (range = 0–30; 0 = completely cognitively impaired, 30 = completely cognitively intact)

^d^BPI-SF-Brief Pain Inventory Short Form (range = 0–10; 0 = no pain, 10 = most pain)

^e^GDS-SF-Geriatric Depression Scale Short Form (range; 0 = no indication of depression, 15 = high possibility of depression)

^f^STAI-Spielberger State or Trait Anxiety Inventory (range; 20 = indicates increased anxiety, 80 = indicates least amount of anxiety)

### Psychophysical results

#### Sensory

Statistically significant sex differences were observed for the stimulus intensity required to evoke mild and moderate pain, with females reporting both mild and moderate pain at lower temperatures than the males (Cohen’s *d* = 0.92 and 0.99, respectively, *p* < 0.01). In contrast, non-painful warmth was perceived at similar temperatures for both sexes (Cohen’s *d* = 0.41, *p* = 0.186). When compared to males, females demonstrated less change in temperature values between their reported warm and mild pain levels (Cohen’s *d* = 0.84, *p* = 0.037; see Table 2).

**Table 2:** Summary of psychophysics of temperature thresholds necessary to produce ratings at each condition

Variables		Min	Max	Median	IQR	*p* value^a^	Effect size^b^
Temperature							
Warmth	Males	31	38	33.0	32.0–33.0	0.186	0.41
	Females	31	35	32.0	32.0–32.8		
Mild pain	Males	34	47	38.5	35.0–44.0	0.002	0.92
	Females	33	39	35.0	34.0–36.0		
Moderate pain	Males	37	48	44.0	40.3–47.3	0.007	0.99
	Females	36	46	38.0	38.0–41.8		
Mild pain > warmth^c^	Males	1.0	15.0	5.0	3.0–10.3	0.037	0.84
	Females	1.0	6.0	2.5	2.0–4.5		
Moderate pain > warmth^c^	Males	5.0	17.0	11.5	7.0–12.0	0.081	0.30
	Females	3.0	13.0	6.0	5.2–10.0		
Unpleasantness							
Warmth	Males	0.0	8.0	0.5	0.0–2.7	0.379	0.30
	Females	0.0	4.5	0.0	0.0–2.2		
Mild pain	Males	0.0	16.0	3.8	0.1–7.3	0.428	0.25
	Females	0.0	5.0	3.5	0.0–5.0		
Moderate pain	Males	2.5	19.0	7.0	5.0–11.8	0.028	0.72
	Females	0.0	9.0	5.0	3.1–7.8		
Mild pain > warmth^d^	Males	−8.0	16.0	3.0	0.1–4.0	0.431	0.76
	Females	0.0	5.0	1.5	0.0–3.0		
Moderate pain > mild pain^d^	Males	−3.0	19.0	6.3	5.0–9.0	0.051	0.78
	Females	0.0	9.0	4.3	2.7–5.0		

Summary of psychophysics of temperature thresholds necessary to produce warmth, mild pain, or moderate pain, and unpleasantness ratings at each condition (age/matched *N* = 24; *n* = 12 male; *n* = 12 female)

^a^ *p* value derived from post hoc Wald *χ* ^2^_(*df* = 1)_ tests of group differences for each condition

^b^Cohen’s *d* for transformed normal data

^c^Difference in degrees Celsius between mild pain and moderate pain

^d^Difference between verbal report of mild pain and moderate pain (0–20 unpleasantness scale)

#### Affective

Ratings of unpleasantness were similar for males and females for both the warmth and mild pain conditions; yet, at the moderate pain intensity level, females provided lower ratings of unpleasantness than males (median 5.0 versus 7.00, Cohen’s *d* = 0.72, *p* = 0.028; see Table 2). This apparent sex difference in the amount of change in reported unpleasantness between mild and moderate pain was confirmed with the statistically significant difference in those values. While not statistically significant, females reported less change in unpleasantness between warm and both mild and moderate pain detection levels than did males with rather large difference effect sizes (Cohen’s *d* = 0.76–0.78; see Table 2).

### fMRI results

#### One-sample results

**Fig. 3:**
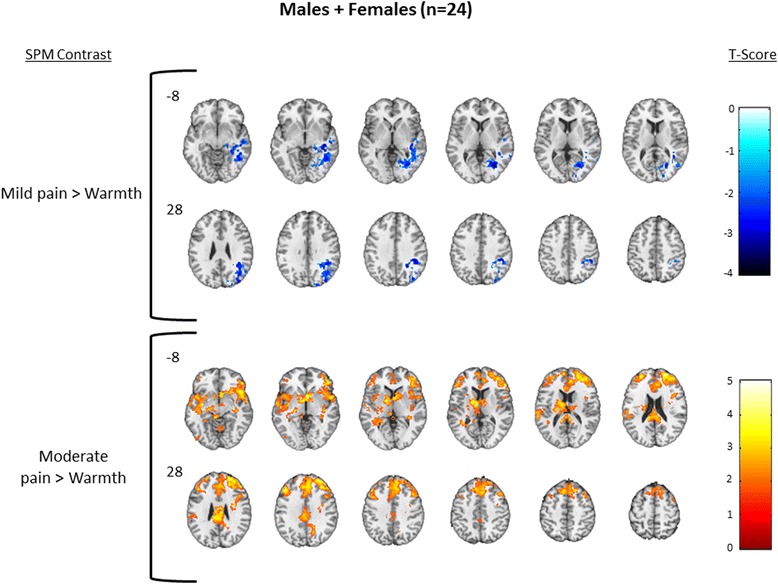
Combined group. One-sample results for the combined group of males + females (*N* = 24, male = 12). Significant clusters were defined as those having a voxel level *p* = 0.05, cluster volume 1659 voxels, and family wise error corrected *p* = 0.05. The *upper row* displays brain deactivation to the contrast of mild pain > warmth, and the *second row* displays brain activation to the contrast of moderate pain > warmth. *Number next to the first image in each row* indicates slice position relative to the AC/PC midline. Axial spacing = 4 mm. *Colorbar* represents T score intensity for each contrast

**Fig. 4:**
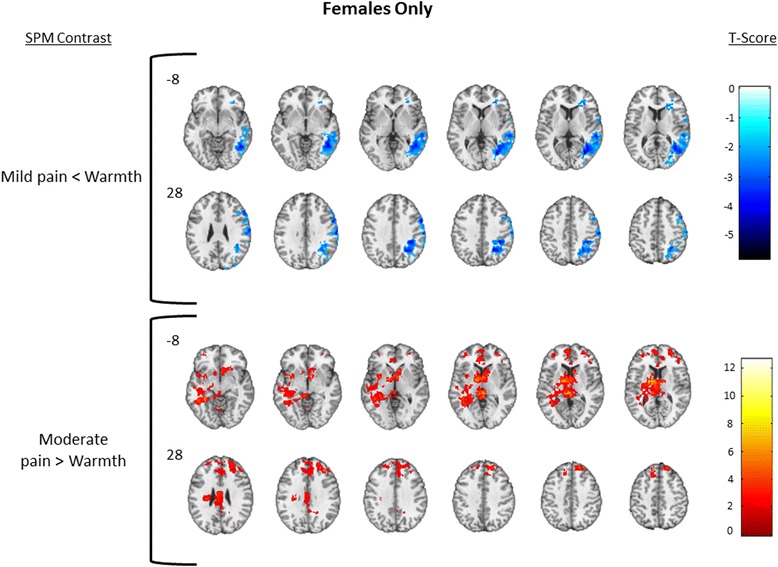
Females only. One-sample results for females only. Significant clusters were defined as those having a voxel level *p* = 0.05, cluster volume 1659 voxels, and family wise error corrected *p* = 0.05. The *upper row* displays brain deactivation to the contrast of mild pain > warmth, and the *second row* displays brain activation to the contrast of moderate pain > warmth. *Number next to the first image in each row* indicates slice position relative to the AC/PC midline. Axial spacing = 4 mm. *Colorbar* represents T score intensity for each contrast

**Fig. 5:**
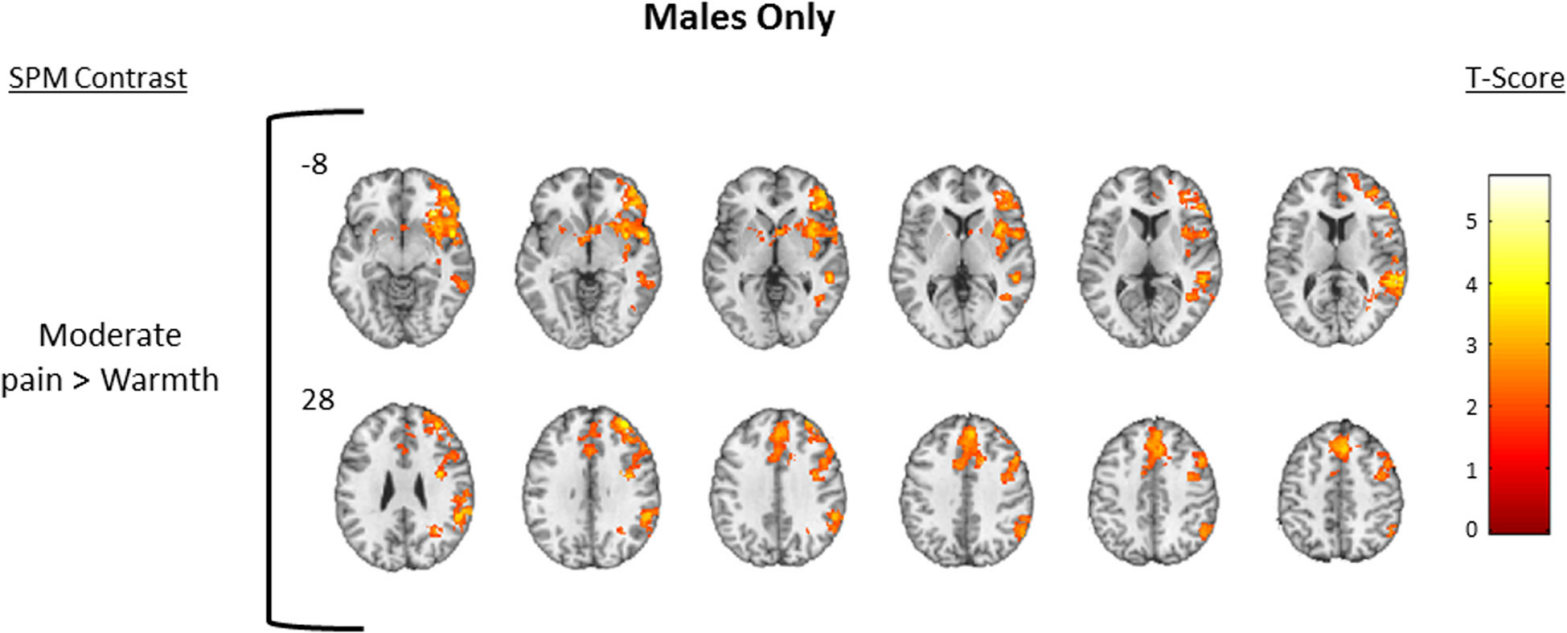
Males only. One-sample results for males only for the contrast of moderate pain > warmth. Significant clusters were defined as those having a voxel level *p* = 0.05, cluster volume 1659 voxels, and family wise error corrected *p* = 0.05. *Number next to the first image in each row* indicates slice position relative to the AC/PC midline. Axial spacing = 4 mm. *Colorbar* represents T score intensity for each contrast

To describe the overall pattern of activation produced by thermal stimulation, we first created one-sample *T* test maps showing brain regions activated during each level of thermal stimulation in the combined sample of males and females. For each of the contrasts mild pain > warmth and moderate pain > warmth, results from one-sample *T* tests for females and males combined (*n* = 24) are shown in Fig. 3; detailed cluster information is shown in Table 3. The combined male + female one-sample contrast of mild > warmth demonstrated deactivation only that was confined to regions associated with core structures in the default mode network (DMN; e.g., cuneus, precuneus, posterior cingulate gyrus (PCG), hippocampus (HIPPO) [37]) and in regions commonly associated with pain (e.g., postcentral gyrus/somatosensory cortex and INS), while the moderate pain > warmth only demonstrated significant activation in regions including cerebellum, fusiform gyrus (FG), and pons. To further describe the within-group patterns of activation, one-sample *T* test maps are shown for female only (*n* = 12) in Fig. 4 and males only (*n* = 12) in Fig. 5; detailed cluster information is shown in Tables 4 and 5, respectively.

**Table 3:** Females and males whole-brain activation (*n* = 24; males = 12)

SPM contrast	Cluster region	Volume	Peak T	MNI coordinates		
	Subregions (BA)	(mm^3^)		*x*	*y*	*z*
Mild pain < warmth	Right cerebrum		−3.96	48	−54	−14
	Superior temporal gyrus	2744		43	−47	17
	Cuneus	2152		18	−79	25
	Precuneus	1623		12	−64	26
	Supramarginal gyrus	1488		42	−44	31
	Middle occipital gyrus	992		31	−65	1
	Dorsal posterior cingulate gyrus	840		23	−64	3
	Right hippocampus	824		20	−30	−3
	Fusiform gyrus (37)	672		47	−54	−18
	Superior temporal gyrus (22)	440		64	−46	3
	Right postcentral gyrus (1,2,3)	328		46	−38	61
	Cingulate cortex (30)	296		20	−67	8
	Inferior temporal gyrus (20)	216		49	−54	−15
	Insula (13)	200		44	−15	3
	Middle temporal gyrus (21)	176		61	−18	−7
Moderate pain > warmth	Left cerebrum		4.03	−32	−84	−30
	Occipital lobe	2384		−35	−77	−18
	Cerebellum	1904		−6	−62	−34
	Left fusiform gyrus	936		−41	−74	−17
	Middle occipital gyrus	928		−42	−71	−15
	Middle frontal gyrus	896		−1	39	31
	Pons	624		13	−41	−38
	Right brainstem	576		6	−44	−45
	Associative visual cortex (19)	392		−35	−84	−14
	Left brainstem	360		−18	−30	−33
	Cerebellum	304		−11	−58	−34
	Lingual gyrus	136		−33	−84	−15

One sample combined males + females analysis. Significant clusters were derived using 3dClustSim and the intrinsic smoothness of the data to define corrected whole-brain cluster thresholds as those having a voxel level *p* = 0.05, cluster volume 1659 voxels, volume >120 mm^3^, and family wise error corrected *p* = 0.05. Areas are reported with the parent cluster first defined by Peak T, followed by subregions within each cluster (MNI coordinates of subregions are approximate). Mild pain < warmth = deactivations; moderate pain > warmth = activations

*SPM* Statistical Parametric Mapping, *BA* Brodmann area, *MNI* Montreal Neuroimaging Institute

**Table 4:** Female-only whole-brain activation (*n* = 12)

SPM contrast	Cluster region	Volume	Peak T	MNI coordinates		
	Subregions (BA)	(mm^3^)		*x*	*y*	*z*
Mild Pain < Warmth	Right cerebrum		−5.76	34	−68	6
	Temporal lobe	30,040		48	−55	−13
	Parietal lobe	17,528		40	−40	39
	Middle temporal gyrus (21)	13,032		61	−31	1
	Superior temporal gyrus (22)	8800		58	−40	8
	Right postcentral gyrus (1,2,3)	4152		62	−18	27
	Right precentral gyrus	3088		58	5	34
	Right fusiform gyrus (37)	2576		47	−52	−19
	Secondary somatosensory cortex (40)	2120		39	−55	42
	Cuneus	1936		23	−88	17
	Precuneus	1880		23	−60	44
	Premotor cortex (6)	1456		59	−6	37
	Somatosensory association cortex (7)	1344		29	−53	46
	Dorsolateral prefrontal cortex (9)	1136		58	5	34
	Primary motor cortex (4)	568		60	−21	44
	Insula	128		41	−28	−3
Moderate Pain > Warmth	Cluster 1: left cerebellum		4.50	−30	−76	−42
	Cerebellum posterior lobe	9984		−28	−55	−41
	Cerebellar tonsil	6432		−9	−55	−42
	Cerebellum anterior lobe	4152		−27	−54	−39
	Right cerebellum	2008		8	−51	−43
	Declive	1632		−28	−79	−29
	Culmen	1296		0	−54	−18
	Dentate	496		12	−47	−33
	Pons	416		−2	−19	−26
	Right brainstem	304		6	−47	−45
	Left brainstem	144		−2	−44	−39
	Cluster 2: left cerebrum		12.65	−16	−6	14
	Right cerebrum	12,064		1	4	1
	Temporal lobe	10,664		−36	−50	5
	Right thalamus	6720		4	−3	8
	Right caudate	3336		15	−12	21
	Superior temporal gyrus	3232		−48	−19	−1
	Caudate head	2160		7	10	4
	Left caudate	2032		−15	−2	15
	Parahippocampal gyrus	1720		−30	−41	−10
	Left insula	1712		−29	−24	18
	Pulvinar	1392		6	−26	3
	Left putamen	1312		−27	−14	10
	Left brainstem	1072		−6	−35	−7
	Middle temporal gyrus	896		−37	−49	3
	Ventral posterior cingulate cortex (23)	880		−2	−30	27
	Insula (13)	824		−42	7	−11
	Right middle cingulate cortex	752		1	−22	29
	Anterior cingulate cortex	616		9	23	−8
	Dorsal posterior cingulate cortex (31)	360		5	−47	31
	Subgenual cingulate cortex (25)	328		3	9	−16
	Cluster 3: frontal lobe		4.67	20	44	44
	Superior frontal gyrus	9072		27	47	23
	Middle frontal gyrus	5560		27	52	16
	Anterior cingulate	3184		12	42	13
	Anterior prefrontal cortex (10)	3000		26	49	28
	Dorsolateral prefrontal cortex (9)	2088		33	36	31
	Dorsal anterior cingulate cortex (32)	1536		−6	35	29
	Ventral anterior cingulate cortex (24)	448		6	29	17

One-sample female-only analysis. Significant clusters were derived using 3dClustSim and the intrinsic smoothness of the data to define corrected whole-brain cluster thresholds as those having a voxel level *p* = 0.05, cluster volume 1659 voxels, volume >120 mm^3^, and family wise error corrected *p* = 0.05. Areas are reported with the parent cluster first defined by Peak T, followed by subregions within each cluster (MNI coordinates of subregions are approximate). Mild pain < warmth = deactivations; moderate pain > armth = activations

*SPM* Statistical Parametric Mapping, *BA* Brodmann area, *MNI* Montreal Neuroimaging Institute

**Table 5:** Male-only whole-brain activation (*n* = 12)

SPM contrast	Cluster region	Volume	Peak T	MNI coordinates		
	subregions (BA)	(mm^3^)		*x*	*y*	*z*
Moderate pain > warmth	Cluster 1: right cerebrum		5.50	40	−2	−22
	Middle frontal gyrus	13,424		6	40	39
	Inferior frontal gyrus	13,152		47	38	−9
	Superior frontal gyrus	9408		1	4	63
	Right insula (13)	6824		35	8	−12
	Superior temporal gyrus (22)	4224		59	−36	21
	Right premotor cortex (6)	4080		16	16	62
	Right supplemental motor area (6)	3440		15	22	62
	Inferior frontal gyrus (47)	2744		50	34	−10
	Dorsolateral prefrontal cortex (9)	2576		45	29	33
	Dorsal anterior cingulate cortex (32)	2392		11	38	21
	Precentral gyrus	1936		55	5	8
	Anterior prefrontal cortex (10)	1632		24	55	27
	Right putamen	1568		24	14	−5
	Temporopolar area (38)	1040		41	23	−25
	Caudate head	560		5	9	−3
	Orbitofrontal area (11)	536		27	25	−15
	Putamen	536		−23	12	−3
	Middle temporal gyrus (21)	184		59	−22	−14
	Subgenual cingulate cortex (25)	152		5	12	−8
	Parahippocampal gyrus (34)	128		34	−4	−23
	Cluster *2*: right cerebrum		5.70	60	−18	20
	Superior temporal Gyrus (22)	6120		58	1	−4
	Middle temporal gyrus (21)	3376		56	8	−18
	Supramarginal gyrus (40)	3256		58	−41	32
	Right postcentral gyrus	2032		58	−20	20
	Insula (13)	536		41	−7	2
	Angular gyrus (39)	368		39	−56	22
	Secondary somatosensory cortex (42)	280		62	−35	20
	Right fusiform gyrus	160		47	−28	−19

One-sample male only analysis. Significant clusters were derived using 3dClustSim and the intrinsic smoothness of the data to define corrected whole-brain cluster thresholds as those having a voxel level *p* = 0.05, cluster volume 1659 voxels, volume >120 mm^3^, and family wise error corrected *p* = 0.05. Areas are reported with the parent cluster first defined by Peak T, followed by subregions within each cluster (MNI coordinates of subregions are approximate)

*SPM* Statistical Parametric Mapping, *BA* Brodmann area, *MNI* Montreal Neuroimaging Institute

#### Between-group comparison of males versus females (males > females, females > males)

**Fig. 6:**
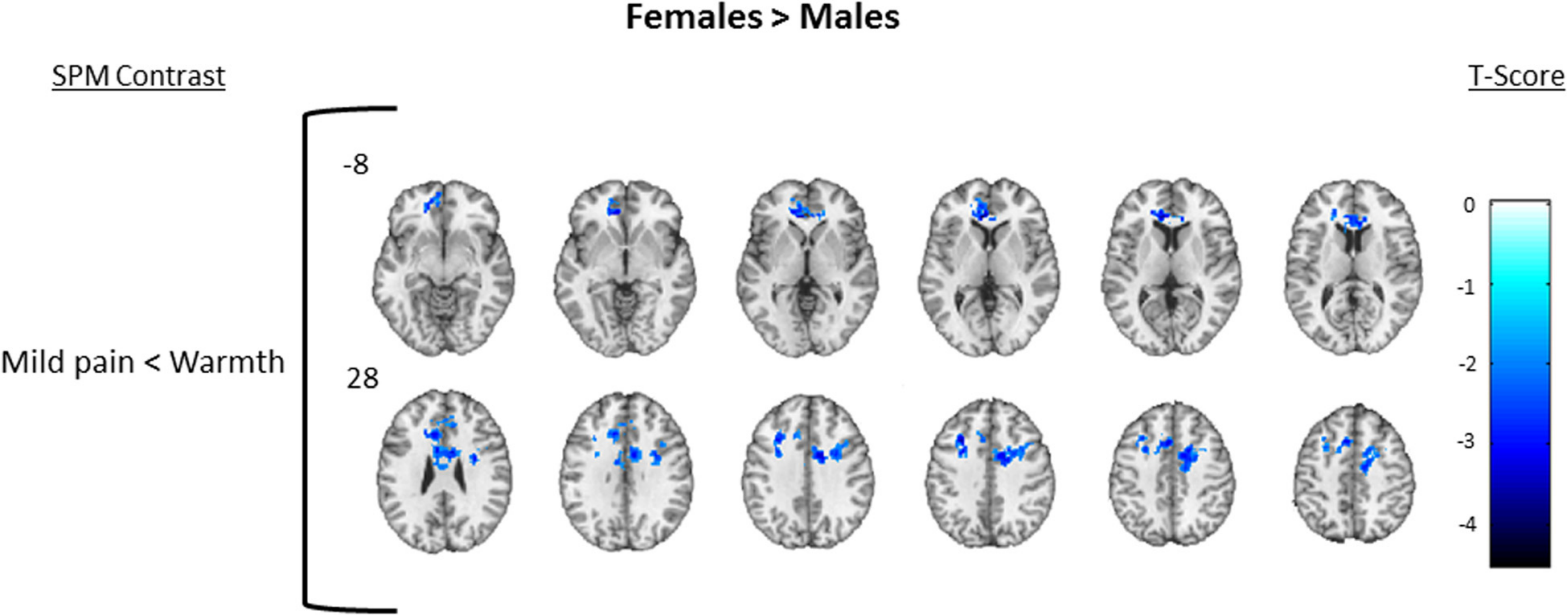
Females > males. Between-group analysis of females > males to the contrast of mild pain > warmth. Significantly deactivated clusters were defined as those having a voxel level *p* = 0.05, cluster volume 1659 voxels, and family wise error corrected *p* = 0.05. *Number next to first image in each row* indicates slice position relative to the AC/PC midline. Axial spacing = 4 mm. *Colorbar* represents T score intensity

**Fig. 7:**
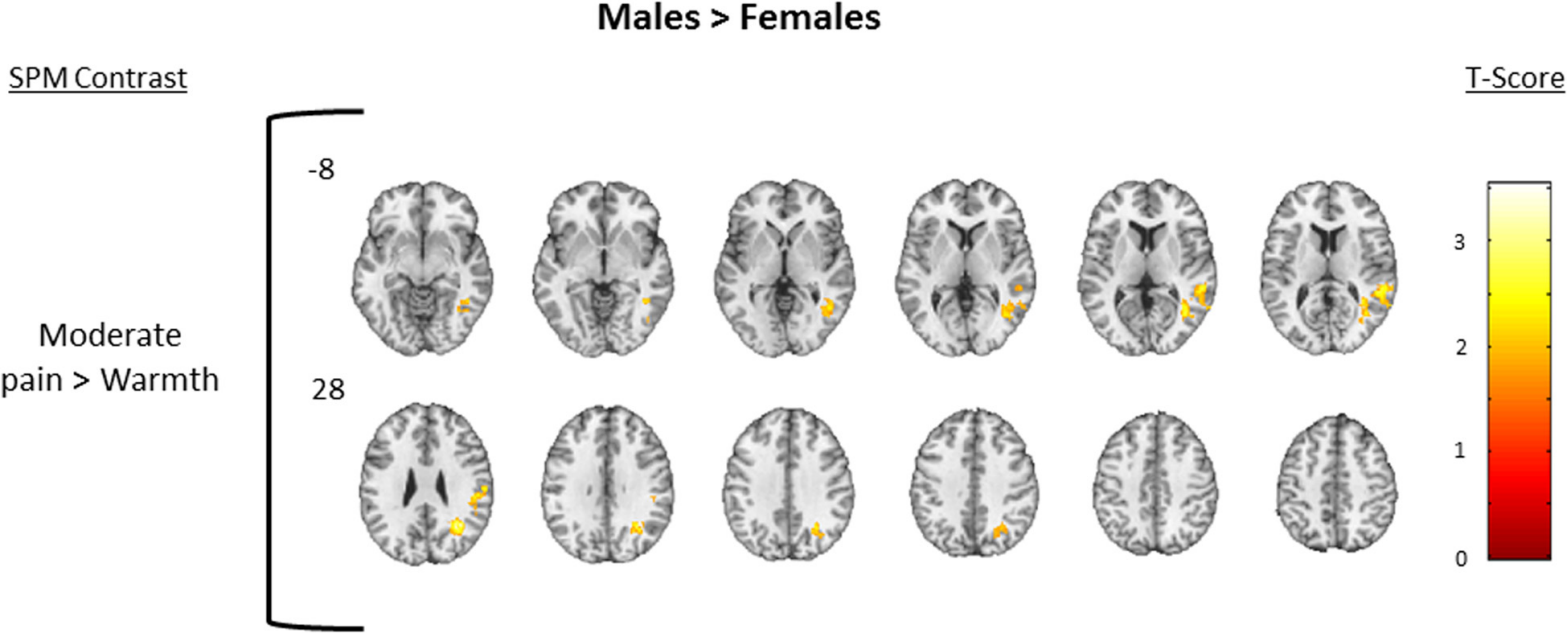
Males > females. Between-group analysis of males > females to the contrast of moderate pain > warmth. Significantly activated clusters were defined as those having a voxel level *p* = 0.05, cluster volume 1659 voxels, and family wise error corrected *p* = 0.05. *Number next to first image in each row* indicates slice position relative to the AC/PC midline. Axial spacing = 4 mm. *Colorbar* represents T score intensity

In the between-group contrasts of females > males, females demonstrated less deactivation in several bilateral brain regions in the mild pain > warmth contrast including the dlPFC, rostral ACC (rACC), and dorsal ACC (dACC) (Fig. 6; Table 6), while in the between-group contrast of males > females, males demonstrated greater activation in several ipsilateral brain regions in the moderate pain > warmth contrast including the pINS, SI, and SII (Fig. 7; Table 6). See Table 7 for MNI coordinates corresponding to pain regions labeled in Figs. 6 and 7. To aid in the interpretation of the findings in Figs. 3, 4, 5, 6, and 7, the pattern of blood oxygenation level dependent (BOLD) signal change for each condition relative to baseline is shown in Additional file 1: Figure S1 and Additional file 2: Figure S2.

**Table 6:** Between-group whole-brain activation (*n* = 24; males = 12)

SPM contrast	Cluster region	Volume (mm^3^)	Peak T	MNI coordinates		
	Subregions (BA)			*x*	*y*	*z*
Mild pain > warmth (deactivation)						
Females > males	Left cerebrum		4.51	−6	34	4
	Limbic lobe	14,608		−8	21	25
	Rostral anterior cingulate cortex (24)	2312		−3	36	5
	Middle frontal gyrus	2280		−25	12	52
	Dorsal anterior cingulate cortex (32)	2192		−7	41	3
	Superior frontal gyrus	1432		−11	11	64
	Left supplemental motor area (6)	912		−13	12	64
	Prefrontal cortex (8)	320		−28	17	42
	Anterior prefrontal cortex (10)	288		−7	49	3
	Dorsolateral prefrontal cortex (9)	256		40	15	38
	Anterior cingulate cortex (33)	120		−5	19	25
Moderate pain > warmth (activation)						
Males > females	Right cerebrum		3.51	32	−58	28
	Superior temporal gyrus (22)	3304		55	−37	9
	Middle temporal gyrus	2472		43	−63	5
	Postcentral gyrus (1,2,3)	712		59	−19	23
	Insula (13)	488		44	−41	19
	Precuneus	464		29	−64	36
	Angular gyrus (39)	352		35	−63	35
	Secondary somatosensory cortex (40)	304		59	−36	22
	Fusiform gyrus (37)	200		48	−41	−18
	Supramarginal gyrus	128		58	−35	24

Between-group analyses. Significant clusters were derived using 3dClustSim and the intrinsic smoothness of the data to define corrected whole-brain cluster thresholds as those having a voxel level *p* = 0.05, cluster volume 1659 voxels, volume >120 mm^3^, and family wise error corrected (FWE) *p* = 0.05. Areas are reported with the parent cluster first defined by Peak T, followed by subregions within each cluster (MNI coordinates of subregions are approximate). Mild pain > warmth, females > males = deactivation (findings result from females being less deactivated than males). Moderate pain > warmth, males > females = activations

*SPM* Statistical Parametric Mapping, *BA* Brodmann area, *MNI* Montreal Neuroimaging Institute

**Table 7:** Correlations of psychophysical reports with activation in common regions located in sensory and affective pain networks (total: *N* = 24, male: *n* = 12, female: *n* = 12)

Region	MNI coordinate	T statistic	Overall associations		Temperature			Affect (unpleasantness)		
	*x*, *y*, *z*		Temp	Affect	Male	Female	*p* value^a^	Male	Female	*p* value^a^
Mild pain < warmth			*r*_s_	*r*_s_	*r*_s_	*r*_s_		*r*_s_	*r*_s_	
			(*p* value)	(*p* value)	(*p* value)	(*p* value)		(*p* value)	(*p* value)	
(L) dlPFC	−4 +32 +34	1.77	−0.05	0.14	0.10	−0.26	0.435	0.11	−0.06	0.719
			(0.833)	(0.515)	(0.771)	(0.448)		(0.754)	(0.868)	
(L) dACC	−6 +44 +1	2.59	−0.24	0.05	−0.05	−0.43	0.384	0.11	−0.29	0.384
			(0.279)	(0.817)	(0.890)	(0.182)		(0.759)	(0.386)	
(L) SMA	−12 +14 +66	2.77	−0.24	−0.12	0.07	0.13	0.897	0.22	−0.44	0.139
			(0.271)	(0.603)	(0.837)	(0.723)		(0.523)	(0.172)	
(L) rACC	−12 +33 +24	2.62	−0.32	0.01	−0.41	−0.01	0.368	*0.34*	*−0.56*	*0.037*
			(0.140)	(0.996)	(0.249)	(0.985)		*(0.307)*	*(0.080)*	
Moderate > warmth										
(R) pINS	+51 −35 +17	2.00	0.02	0.26	0.06	−0.20	0.576	0.17	0.20	0.944
			(0.936)	(0.230)	(0.865)	(0.549)		(0.621)	(0.556)	
(R) SI	+60 −21 +28	2.28	0.01	0.37	0.01	−0.37	0.395	0.20	0.04	0.726
			(0.995)	(0.085)	(1.000)	(0.256)		(0.591)	(0.916)	
(R) SII	+60 −34 +21	1.99	0.07	*0.43*	0.28	−0.27	0.230	0.51	0.37	0.711
			(0.765)	*(0.043)*	(0.423)	(0.419)		(0.140)	(0.262)	

Values in each cell are *beta* and (*p* value). Entries in italics indicate either statistically statistic or meaningful effect sizes and respective differences

*MNI* Montreal Neurological Institute, *L* left, *R* right, *dlPFC* dorsolateral prefrontal cortex, *dACC* dorsal anterior cingulate cortex, *SMA* somatomotor area, *rACC* rostral anterior cingulate cortex, *pINS* posterior insula, *SI* primary somatosensory cortex, *SII* secondary somatosensory cortex

^a^ *z* test of independent correlations

### Associations between activation and psychophysical results

**Fig. 8:**
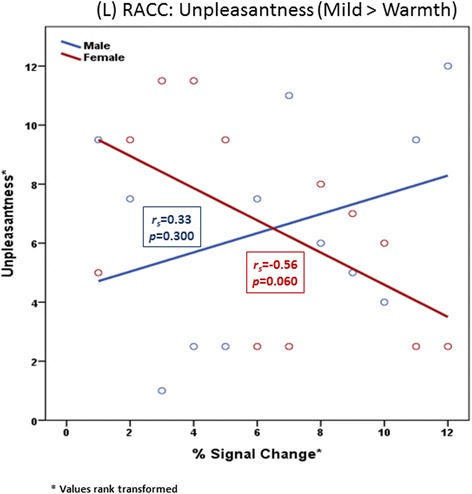
Rostral anterior cingulate cortex. Sex differences in the association between unpleasantness reported and percent signal change values in the rostral anterior cingulate cortex (rACC) for the condition of mild pain > warmth

**Fig. 9:**
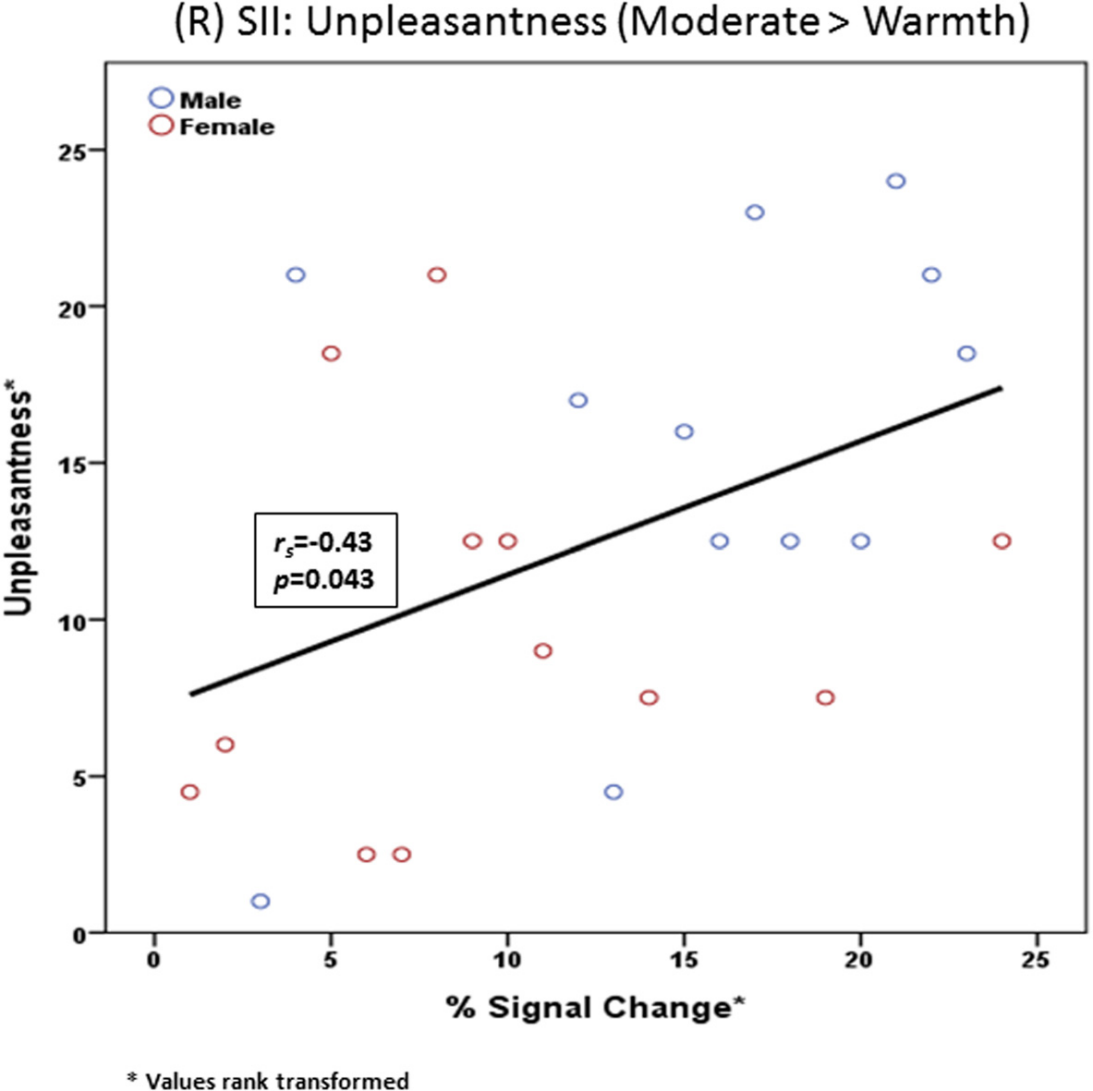
Secondary somatosensory cortex. Overall group association between unpleasantness reported and percent signal change values in the contralateral secondary somatosensory (SII) cortex for the condition of moderate pain > warmth

To explore and describe the perceptual importance of significant sex differences in brain activations, we examined the associations of the BOLD signal change values in each of the ROIs above showing significant sex differences with psychophysical self-reports. Summaries of these associations for all 24 participants and for each sex (*n* = 12 in each group) are described in Table 7. Signal change values for each ROI are presented in Figs. 8 and 9.

#### Mild pain > warmth

**Fig. 10:**
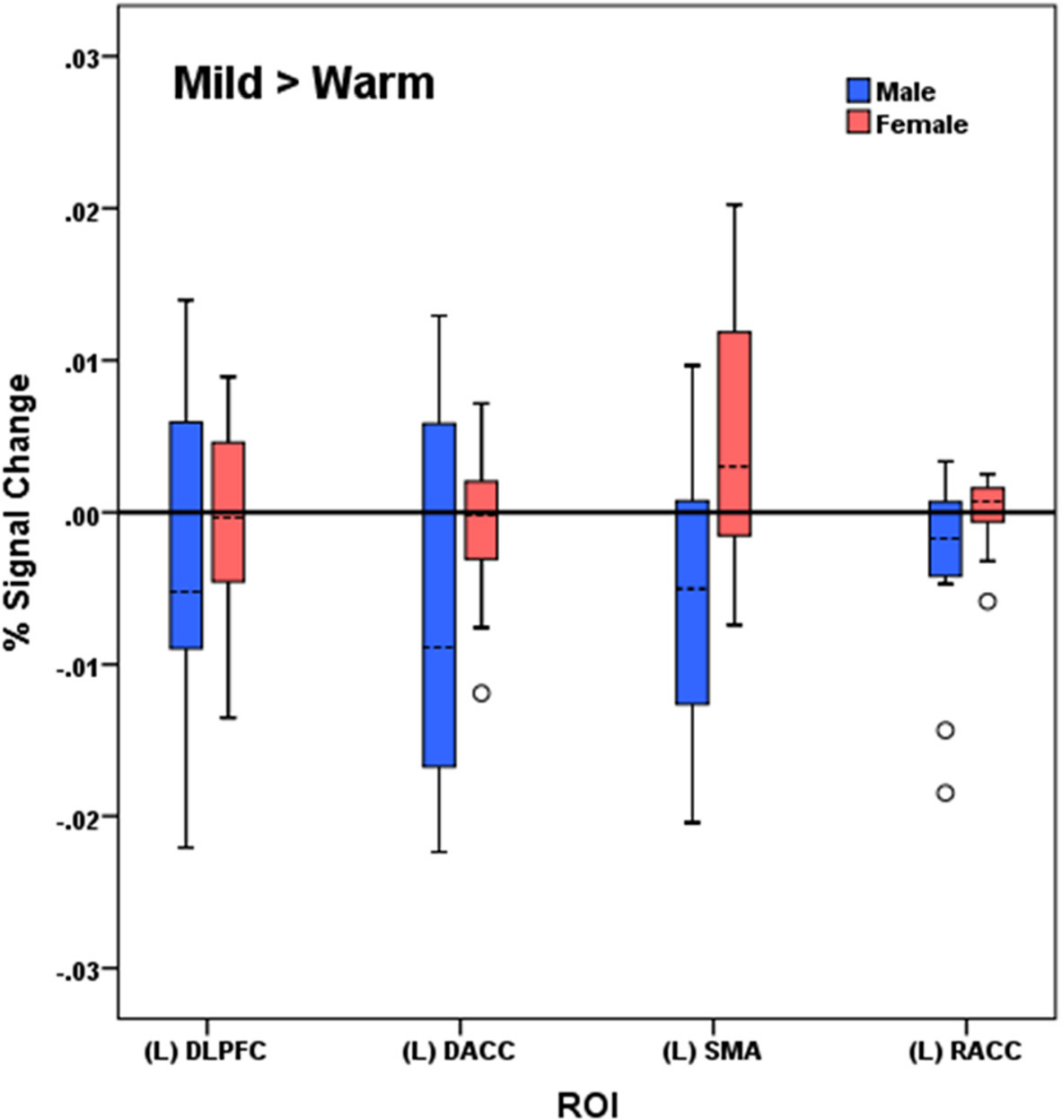
Mild > warm percent signal change values by sex. Between-group percent signal change values by sex for pain regions of interest in the mild > warm contrast condition. *L*=left, *DLPFC*=dorsolateral prefrontal cortex, *SMA*=somatomotor area, *RACC*=rostral anterior cingulate cortex

A statistically significant and contrasting pattern of effect sizes by sex for the associations of deactivation levels with perceived unpleasantness was observed for the left (contralateral) rACC region (males: *beta* = 0.34 or positive relationship; females: *beta* = −0.56 or inverse relationship; two-tailed *z* = 2.09, *p* = 0.037). Higher levels of deactivation in left rACC were associated with less change in unpleasantness between warmth to mild pain in females but not so for males. Within the left rACC, higher levels of deactivation tended to be associated with greater increases in unpleasantness from warmth to mild pain for males (Fig. 10). No other statistically significant associations were observed.

#### Moderate pain > warmth

**Fig. 11:**
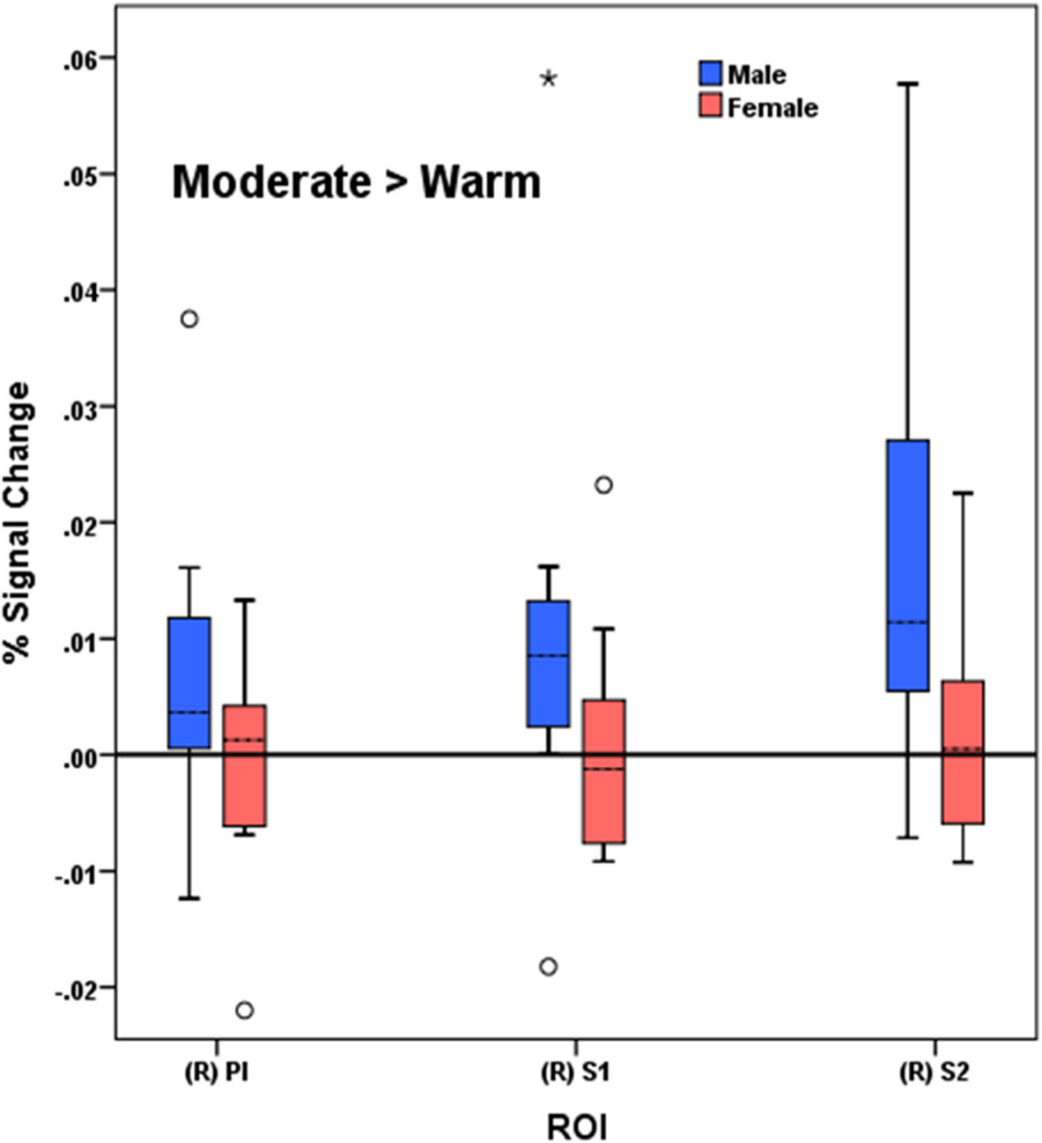
Moderate > warm percent signal change values by sex. Between-group percent signal change values by sex for pain regions of interest in the moderate > warm contrast condition. *pINS* posterior insula, *SI* primary somatosensory cortex, *SII* secondary somatosensory cortex

Greater BOLD signal changes in right (ipsilateral) SII were associated with greater increases in self-reported unpleasantness ratings between the moderate pain and warmth conditions across both females and males (*beta* = 0.43, *p* = 0.043; Fig. 11). No other associations or tests of sex differences in regions activated during the moderate > warmth condition were statistically significant.

## Discussion

To our knowledge, this is the first study to report sex differences in pain perception and brain response to experimental thermal pain in older adults. Our first hypothesis (psychophysical), that when compared to older males, older females would report “mild” and “moderate” pain at lower stimulus intensities (i.e., be more pain sensitive) and would report greater perceived unpleasantness, was partially supported. While older females were more sensitive to pain (i.e., they required lower stimulus temperatures to detect mild and moderate pain intensity), they did not report greater perceived pain unpleasantness. Our second hypothesis (neurophysiological) that, when compared to older males, older females would exhibit greater brain activations in the sensory/discriminative, affective/motivational, and cognitive/evaluative pain networks, was also partially supported. Interestingly, older females demonstrated less deactivation to mild pain in cognitive/evaluative and affective/motivational pain networks. Conversely, while both groups demonstrated only activations to moderate pain, males demonstrated greater activation to moderate pain in the sensory/discriminative pain network. Our overall finding of greater deactivation to mild pain and greater activation to moderate pain are supported by a study in which the relationship and pattern of activations and deactivations to “low” and “high” pain were explored [38]. Kong and colleagues found that participants exhibited greater deactivations in response to “low” pain but exhibited greater activations to “high” pain. These researchers concluded that each of these patterns of signal change (deactivation and activation) contribute to different aspects of the pain experience [35]. For example, varying brain signal changes with activations and deactivations may be associated with an organisms’ attempt to evaluate the threat occurring with intense pain or alternatively signal changes could be related to the cognitive load occurring with pain [35]. It is plausible that brain deactivations could be related to pro-nociception or activation of anti-nociceptive mechanisms. A seminal study examining the DMN in response to block stimulus events (visual and auditory) found that spontaneous activity in DMN regions occurred during both task and rest and that greater DMN activity during stimulus was associated with greater activation in sensory regions [39]. While many pain studies use paradigms to help ensure that subjects attend to pain [40], studies of passive pain tasks may help uncover the role of the DMN during the experience of mild pain compared to more noxious levels of pain. The clinical importance of attending to mild pain cannot be underestimated. Persistent mild pain may be a first sign of an impending clinical problem requiring medical attention.

With regard to sex-effects on the detection of thermal pain stimuli, our results are in partial agreement with psychophysical studies in younger adults showing that, when compared to younger males, younger females demonstrate significantly lower pain thresholds (increased sensitivity) to both mild and moderate pain (reviewed in [6]). The differences in sensory sensitivity to thermal pain stimuli (i.e., perceptual thresholds) in our sample of older males and females were similar to those previously reported in younger adults. However, in contrast to findings from studies of younger adults (where females generally show greater pain-associated unpleasantness than males; reviewed in [6]), we did not find significant sex differences in pain-related affective responses (measured as stimulus unpleasantness) between males and females, that is, we did not find evidence for increased affective responses to thermal pain in older females (relative to older males) and in fact we found that older females found pain to be less unpleasant.

In this sample of older adults, we did find similar sex-associated differences in brain-activation patterns demonstrated in younger individuals. When compared to older males, older females demonstrated greater activation in PFC [20, 21] and ACC [41]. Conversely, and similar to younger individuals, when compared to older females, older males demonstrated increased activation in the SI and SII cortices [41]. Despite these similarities, sex differences in the response to thermal pain in older adults were noted. Older females exhibited lower pain detection temperatures than men (increased pain sensitivity) for both mild and moderate pain, suggesting a greater ability to detect and identify painful stimuli than their male counterparts. However, in general, this increased sensitivity to pain was not reflected in greater activation in the sensory/discriminative pathway but rather via less deactivation in key regions of the DMN (cuneus, precuneus, PCC, HIPPO), somatomotor area (SMA), and in components of both the affective/motivational (rACC, dACC) and cognitive/evaluative networks (dlPFC). While not generally regarded as part of the “pain matrix,” the SMA demonstrates functional connectivity with several regions implicated in sensory (e.g., pINS, SI, SII) and affective (e.g., ACC) pain processing (reviewed in [42]).

Though the precise network of pain processing has yet to be determined, the pattern of less deactivation to mild pain and increased sensitivity to overall pain observed in females in the current study could possibly be attributed to decreased efficiency of endogenous opioid systems in aging [43]. Animal studies demonstrate age-associated declines in endogenous opioid system function [44, 45], and these findings appear to extend into humans. Using psychophysics, Washington and colleagues showed that when compared to younger people and in response to similar pain, older people demonstrated a significantly lower endogenous analgesic response concluding that age-associated declines in endogenous opioid systems place older adults at risk for reduced ability to cope with pain [46]. A recent positron emission tomography (PET) found that both gender and age have an effect in central mu-opioid receptor binding. Mu-opioid receptor binding was found to increase with age in neocortical areas and the putamen. Interestingly, when compared to males, pre-menopausal females demonstrated greater central mu-opioid receptor binding in multiple cortical and subcortical regions. However, in post-menopausal females, such as those in the current study, mu-opioid receptor binding decreased to levels below those of men [47]. Considerable evidence supports the role of central mu-opioid receptor activity in endogenous pain control, and several PET studies have shown mu-opioid receptor activation during acute pain in regions demonstrating deactivation in females in the current study (e.g., ACC and PFC [48–50]). Furthermore, pain-induced activation of the mu-opioid receptor (via endogenous opioid system) was found to negatively correlate with both sensory and affective ratings of pain [50, 51]. In the current study, sex differences in the association between BOLD signal change and affective pain reports were noted in the left (contralateral) rACC with older males demonstrating positive associations between BOLD signal change and reports of unpleasantness, while older females demonstrated negative associations between BOLD signal change and reports of unpleasantness. Interestingly, we found that among the entire sample of older adults, reported unpleasantness associated with moderate pain was associated with brain signal change in right (ipsilateral) SII. Though the ACC is well known to encode pain affect [52, 53], findings from the current study elevate both the rACC and SII as regions responsible for processing pain affect in older adults.

Contrary to our hypotheses, males exhibited greater activation to moderate pain in lateral (sensory/discriminatory) pain pathway regions than did females (e.g., pINS, SI, and SII). The pINS, SI, and SII collectively function to encode pain intensity and sustained attention to pain [16, 54]. Greater activation in the lateral pain system might be due in part to the greater stimulus intensities (higher temperatures) identified by males as reflecting “mild” and “moderate” pain. Decreased quantity and quality of peripheral a-delta nociceptive fibers have been suggested as a contributory factor leading to increased sensory thresholds in aging [55], and these sex differences in temperature detection thresholds could be the result of greater reductions in epidermal sensory fibers in older males when compared to older females [56]. Although speculative, such differences might result in males requiring greater thermal stimulus intensities to detect pain and consequently exhibit greater brain activation in sensory pain regions.

### Limitations

Because of the small sample size, the interpretation of significant sub-activations in each cluster is assumed based on cluster reports produced via our imaging analyses. As noted by Woo and colleagues [35], the precise location of regions identified in a large activation cluster is difficult to ascertain. Based on further recommendations from Woo and colleagues [35], we report the peak T and MNI coordinates of the largest regions within each parent cluster followed by the approximate subcluster peak activations. We determined subcluster peak activations by examining for the activation of maximum activation within each subcluster. Since the larger study from which this sub-sample was drawn included people with dementia, our design and methods for examining psychophysics and brain activation were influenced by the protection of cognitively impaired human subjects. Due to this focus on protection, we used a somewhat non-conventional approach to psychophysics in that we used individual percepts in our model and did not measure severe pain or pain tolerance. Thus, careful attention should be made when comparing the current study findings with other studies. Nonetheless, findings from this study add to the very limited body of psychophysical and neurophysiological research on pain in older adults.

## Conclusions

In summary, the current study found that elevated sensory pain detection levels observed in younger females extends into older females. However, past findings in younger populations that females rate comparable painful stimulus intensities as more unpleasant than do males did not extend into the older population. Interestingly, both males and females demonstrated deactivation in response to mild pain with females demonstrating significantly less deactivation in key regions typically associated with the DMN and pain affect. The DMN is generally posited to engage neural systems that are involved in the monitoring of the “external environment” (e.g., what external threats exist) or invoking an “internal awareness” (e.g., “what is happening to me?”; reviewed in [37]). Each of these suppositions could have clear indications for individuals experiencing pain. It is important to note that the mild and moderate intensity measures used in the current study would not necessarily evoke a life-threatening feeling.

Current findings further advance the DMN as a system that may mitigate the pain experience and the INS and SII as key regions potentially involved in both sensory and affective pain processing in older adults [43]. Moreover, when compared to males, post-menopausal older females may have altered endogenous opioid systems, which place them at an increased risk of chronic pain and pain-related disability (e.g., avoidance, functional decline). Indeed, reduced baseline pain sensitivity coupled with altered endogenous analgesic systems seems to place individuals at risk for the development of chronic pain (reviewed in [57]). Future directions are to explore the functional connectivity between the DMN, periaqueductal gray (PAG), aINS, and pINS and key brain structures involved with cognitive, evaluative, and modulatory pain processing in older adults. Additionally, studies examining the role of activations and deactivations to thermal pain responses across a wide range of ages may further assist in the interpretation of age- and sex-associated differences in pain processing.

## Additional files

Additional file 1: Figure S1. Females only. One-sample results for females only. Significant clusters were defined as those having a voxel level *p* = 0.05, cluster volume 1659 voxels, and family wise error corrected *p* = 0.05. The upper row displays brain deactivation to the contrast of mild pain > baseline, and the second row displays brain activation to the contrast of moderate pain > baseline. Number next to the first image in each row indicates slice position relative to the AC/PC midline. Axial spacing = 4 mm. Colorbar represents T score intensity for each contrast. (TIF 443 kb) Additional file 2: Figure S2. Males only. One-sample results for males only. Significant clusters were defined as those having a voxel level *p* = 0.05, cluster volume 1659 voxels, and family wise error corrected *p* = 0.05. The upper row displays brain activation to the contrast of mild pain > baseline, and the second row displays brain activation to the contrast of moderate pain > baseline. Number next to the first image in each row indicates slice position relative to the AC/PC midline. Axial spacing = 4 mm. Colorbar represents T score intensity for each contrast. (TIF 389 kb)

## Abbreviations

a: anterior
ACC: anterior cingulate cortex
BOLD: blood oxygenation level dependent
BPI: Brief Pain Inventory
d: dorsal
dl: dorsolateral
DM: dorsomedial
DMN: default mode network
DRS: Dementia Rating Scale
EPI: echo planer image
f: functional
FC: functional connectivity
FG: fusiform gyrus
FOV: field of view
FWHM: full-width half-maximum
GDS: Geriatric Depression Scale
GLM: general linear model
HIPPO: hippocampus
INS: insula
IQR: inter-quartile range
MMSE: Mini-Mental State Exam
MNI: Montreal Neurological Institute
MRI: magnetic resonance imaging
p: posterior
PAG: periaqueductal gray
PCG: posterior cingulate gyrus
PET: positron emission tomography
PFC: prefrontal cortex
r: rostral
ROI: region of interest
SES: socioeconomic status
sg: subgenual
SI: primary somatosensory
SII: secondary somatosensory
SMA: somatomotor area
STAI: State-Trait Anxiety Inventory
T: Tesla
TE: echo time
TFE: time of flight echo
TR: time to repeat
VPL: ventral posterolateral
