# Supplementary figures and images for: Sex differences in psychophysical and neurophysiological responses to pain in older adults: a cross-sectional study
Source: Biol Sex Differ. 2015 Nov 16;6:25. doi: 10.1186/s13293-015-0041-y (PMC4647695; doi:10.1186/s13293-015-0041-y)

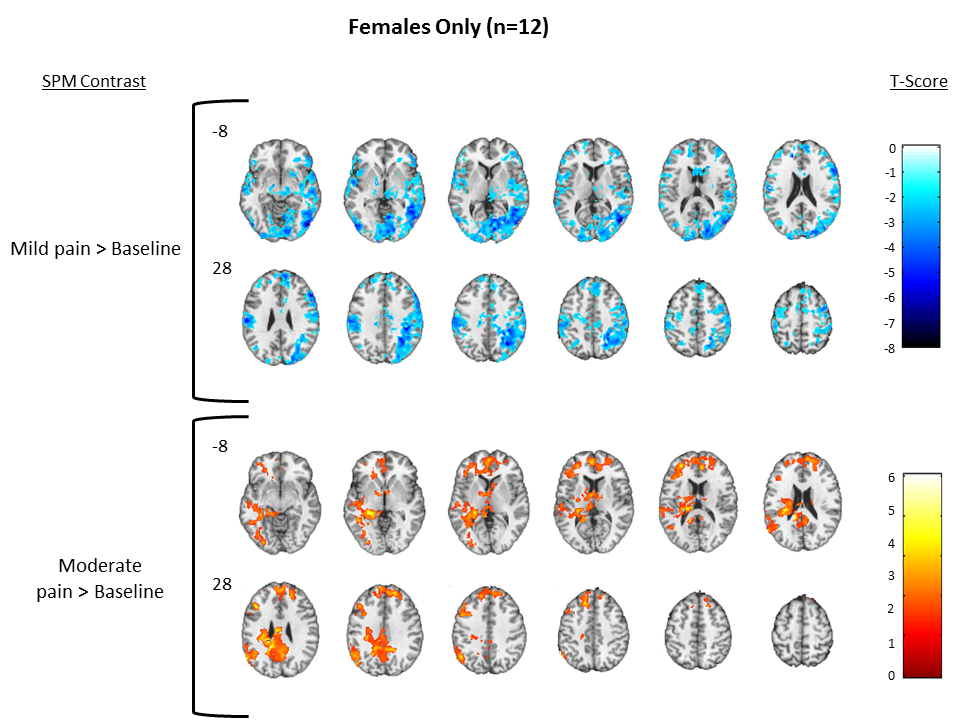

Supplement: Additional file 1: Figure S1. — Females only. One-sample results for females only. Significant clusters were defined as those having a voxel level p = 0.05, cluster volume 1659 voxels, and family wise error corrected p = 0.05. The upper row displays brain deactivation to the contrast of mild pain > baseline, and the second row displays brain activation to the contrast of moderate pain > baseline. Number next to the first image in each row indicates slice position relative to the AC/PC midline. Axial spacing = 4 mm. Colorbar represents T score intensity for each contrast. (TIF 443 kb) [file 13293_2015_41_MOESM1_ESM.tif]

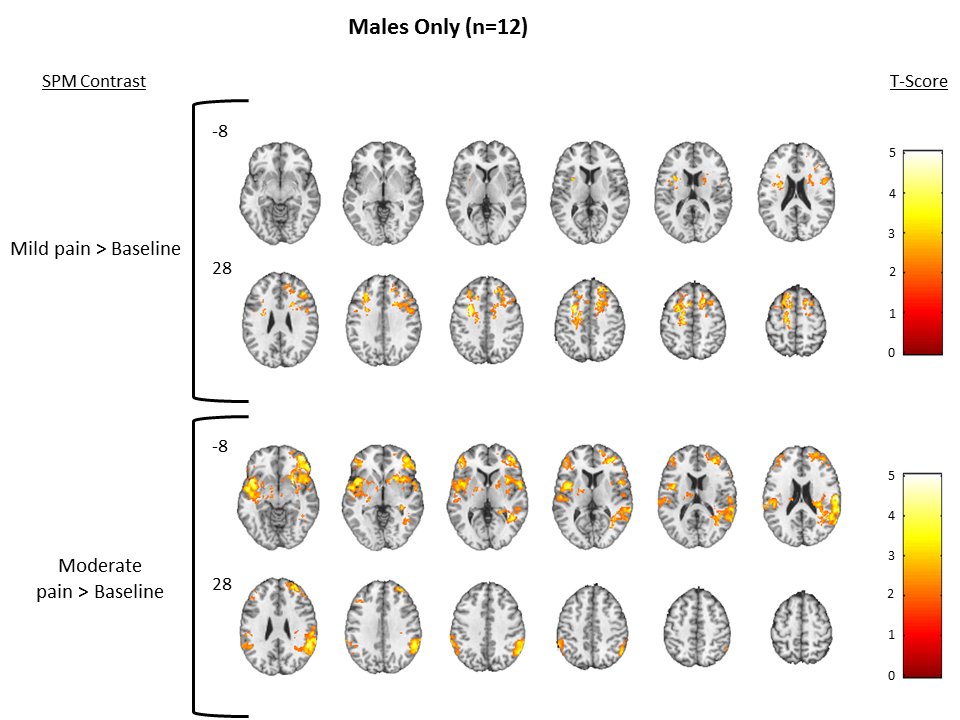

Supplement: Additional file 2: Figure S2. — Males only. One-sample results for males only. Significant clusters were defined as those having a voxel level p = 0.05, cluster volume 1659 voxels, and family wise error corrected p = 0.05. The upper row displays brain activation to the contrast of mild pain > baseline, and the second row displays brain activation to the contrast of moderate pain > baseline. Number next to the first image in each row indicates slice position relative to the AC/PC midline. Axial spacing = 4 mm. Colorbar represents T score intensity for each contrast. (TIF 389 kb) [file 13293_2015_41_MOESM2_ESM.tif]
